# Surface acoustic wave actuated plasmonic signal amplification in a plasmonic waveguide

**DOI:** 10.1186/s11671-023-03951-0

**Published:** 2024-01-09

**Authors:** Rohit Gupta, Kuntal Barman, Liang-Yun Lee, Anuj Chauhan, Jian-Jang Huang

**Affiliations:** 1 Graduate Institute of Photonics and Optoelectronics, National Taiwan University, Taipei, 10617 Taiwan; 2 Department of Electrical Engineering, National Taiwan University, Taipei, 10617 Taiwan

**Keywords:** Surface plasmon polaritons, Plasmonic waveguide, Inter-digital transducer, Surface acoustic wave

## Abstract

Enhancement of nanoscale confinement in the subwavelength waveguide is a concern for advancing future photonic interconnects. Rigorous innovation of plasmonic waveguide-based structure is crucial in designing a reliable on-chip optical waveguide beyond the diffraction limit. Despite several structural modifications and architectural improvements, the plasmonic waveguide technology is far from reaching its maximum potential for mass-scale applications due to persistence issues such as insufficient confined energy and short propagation length. This work proposes a new method to amplify the propagating plasmons through an external on-chip surface acoustic signal. The gold–silicon dioxide (Au-SiO_2_) interface, over Lithium Niobate (LN) substrate, is used to excite propagating surface plasmons. The voltage-varying surface acoustic wave (SAW) can tune the plasmonic confinement to a desired signal energy level, enhancing and modulating the plasmonic intensity. From our experimental results, we can increase the plasmonic intensity gain of 1.08 dB by providing an external excitation in the form of SAW at a peak-to-peak potential swing of 3 V, utilizing a single chip.

## Introduction

Due to the rapid scaling of electronic devices, electronic interconnects are reaching their limit in switching speed and signal loss. Therefore, optical on-chip-interconnects are essential for future high-speed nano-integrated circuits. Traditional light propagation is constrained by the diffraction limit of optical waveguides [1, 2]. It severely hinders the feasibility of nanoscale on-chip interconnection. Three kinds of nanoscale waveguides are available for optical confinement: photonic crystal based [3, 4], ultra-high index-contrast nanowires [5–7], and surface plasmon (SP) waveguides [8, 9]. Among the above, SP waveguides are superior to the other two regarding confinement efficiency. Conceptually, nanoscale confinement can be achieved by introducing materials with negative permittivity [10]. Noble plasmonic metals are well-known materials with negative permittivity, popularly known for generating and propagating surface plasmon polariton (SPP) modes [11, 12]. SPPs are excited by incident light on a noble metallic or semi-metallic surface. The excited electrons on the metal surface undergo periodic oscillation, resulting in an electromagnetic wave propagating along metal–semiconductor interfaces [13, 14].

Over the last few decades, several plasmonic waveguide structures have been modeled theoretically and demonstrated experimentally. The fundamental structures of plasmonic waveguides, including metal V-shape grooves [15], wedges [16], ring resonators [17], and nanowires [18], exhibit decent confinement but with tradeoffs of high propagation loss along the metal interface. Therefore, researchers have developed a hybrid plasmonic waveguide, which combines the properties of a silicon slab waveguide and surface plasmon, to improve the propagation length of up to several hundred micrometers. Depending on the architecture, several plasmonic waveguides are available in the literature, such as plasmonic slot waveguides [19], which are popular in the nanoscale confinement of plasmonic modes. The typical slot structures are generally based on the metal–semiconductor-metal (MSM) [19–21] or metal–insulator-metal (MIM) [22–26] configuration. The dimensions of the sandwiched slot structure play an essential role in the selectivity of propagating modes.

The MSM-based plasmonic slot waveguides exhibit excellent mode coupling. The photocurrent generated by the SP wave in MSM-based plasmonic waveguides can also be effectively employed for signal transmission [27]. An infrared (IR) photodetector is developed based on incorporating Hg-Te quantum dots with a plasmonic waveguide. A graphene-based hybrid plasmonic structure is studied by Zhu et al., [28] where an ultra-small modal area of the order of 10^–4^ λ^2^ (λ is the optical wavelength) and longer propagation length can be achieved by optimizing geometric structural parameters and the chemical potential of graphene with negligible cross-talk [29]. In another study of a graphene-based plasmonic waveguide, a modulator is developed with periodic grating-based perpendicular gates that vary the potential at different locations of the waveguide to alter the Femi level [30]. The plasmonic nanoparticle-chain-based waveguides are also popular for nanoscale confinement, where the nanoparticle diameter is used to select the appropriate propagating mode [31]. In addition, a comparative study between nanoparticle-chain-based, nanostrip, and slot hybrid plasmonic waveguides was conducted, where they showed that the plasmonic modes supported by metal nanoparticle chain waveguides provide the highest in-plane enhancement [32]. However, the nanoparticle-based waveguide suffers from higher propagation loss. A tradeoff between plasmonic energy confinement and propagation length has to be made, and it is dependent on the effective refractive index. Therefore, an external process for improving and modulating the confined energy (without changing the structure) is beneficial.

We previously demonstrated a method to enhance the plasmonic response of gold nanoparticles utilizing plasmon–phonon interactions [33]. With surface acoustic wave (SAW) modulation, we induce a sustained drag effect on the plasmons, resulting in a redistribution of the electron cloud on the nanoparticle surface. The interaction between SAW and plasmons reduces the effective energy gradient and enhances the electron density of the plasmonic state. The modulation of plasmonic relaxation damping through SAW allows for improved control over the plasmon's energy loss and return to the ground state after excitation.

In this work, we propose a SAW integrated hybrid plasmonic waveguide structure for on-chip plasmonic signal enhancement. The proposed active plasmonic device is analogous to a repeater in the traditional communication system. The structure comprises a semiconductor–metal-insulator-semiconductor (SMIS) based hybrid plasmonic waveguide integrated with an interdigital transducer (IDT) for SAW generation. SAW can effectively interact with plasmonic waveguide modes and enhance transmission quality and fidelity. Moreover, SAW can be modulated by applying a voltage to the IDT, which provides excellent voltage tuning capability to the plasmonic device. Therefore, the waveguide can attain a different range of plasmonic confinement and propagation distance by changing SAW resonant characteristics.

## Device fabrication and experimental setup

### Materials and methods

**Fig. 1:**
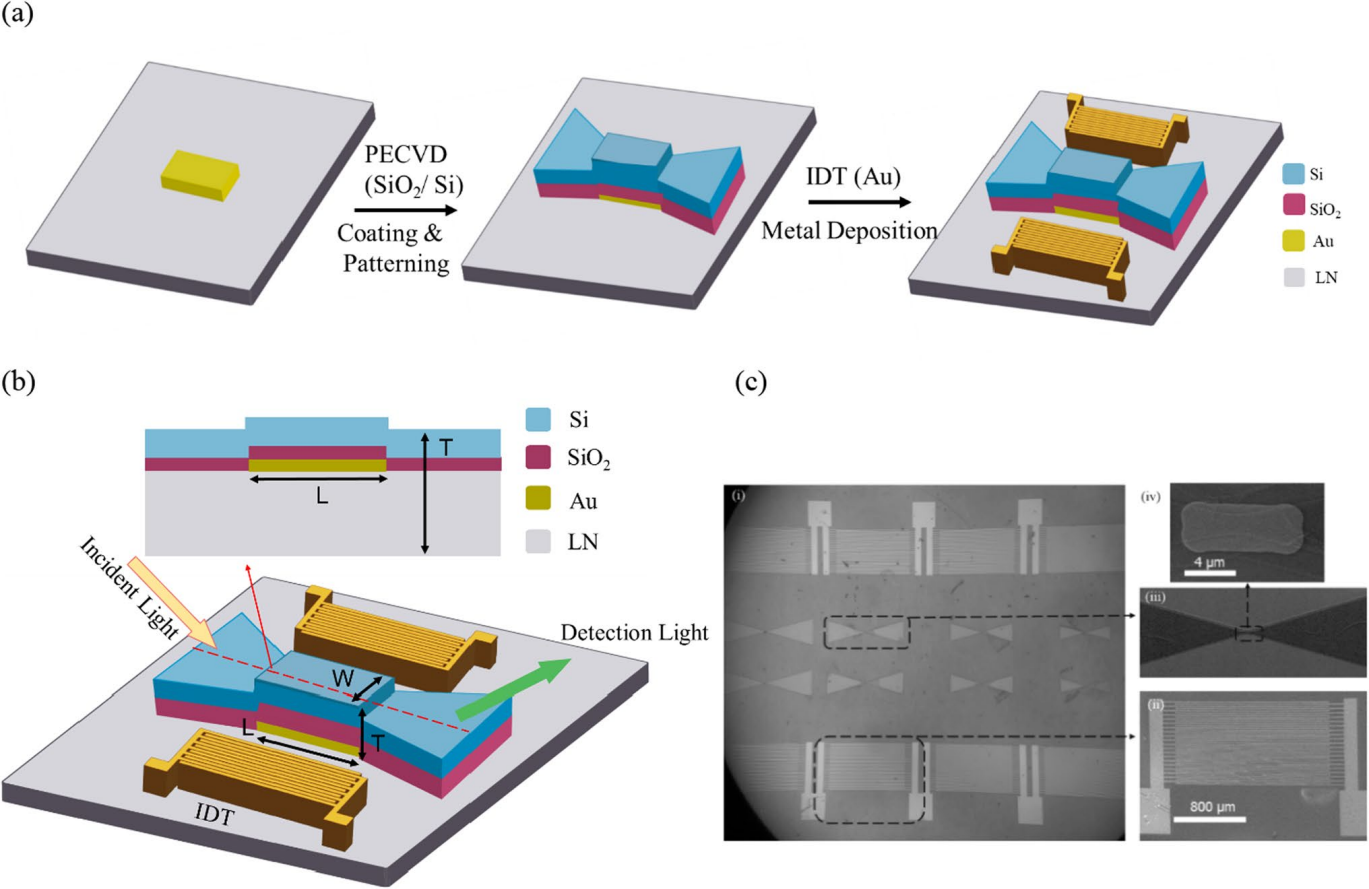
**a** Schematic steps of device fabrication. **b** Schematic of the plasmonic waveguide structure integrated with IDTs. The light was incident from the left and collected from the right side of the waveguide. The acoustic waves generated from the IDT were impeding from one side of the channel. The broken red line denotes the side view of the structure and is illustrated in the inset. **c** SEM image of the device on the LN substrate. (i) the full image of the device taken through an optical microscope, (ii)–(iii) zoom-in view of the IDT and waveguide, (iv) further zoom-in of the top-view of the waveguide

Figure 1a schematically shows the steps of device fabrication. We first deposited Ti/Au (10/30 nm) metal layers on the X-cut Lithium Niobate (LN) substrate by e-beam evaporator. The metal stack acts as the waveguide channel. A 50 nm -thick SiO_2_ film and a 300 nm -thick Si layer were deposited by plasma-enhanced chemical vapor deposition (PECVD) and patterned by RIE (reactive ion etching) to form the waveguide structure. IDTs were fabricated by depositing Ti/Au (20/200 nm) films on the LN substrate. The device consists of 40 fingers, each with a width of 12.5 µm and is 12.5 µm apart. The designated target resonant frequency is 750 MHz [34]. Figure 1b shows the 3-dimensional device structure consisting of a plasmonic waveguide and two IDTs. The post-fabrication scanning electron microscope (SEM) images are shown in Fig. 1c. The waveguide channel width (W) is fixed at 2 µm to ensure consistent optical flux injected to the plasmonic waveguide and to obtain a high-quality SPP response for the study. The channel length (L) is selected as 2, 4, 6, and 8 µm for analyzing SPP propagation and its interaction with SAWs.

### Experimental setup

**Fig. 2:**
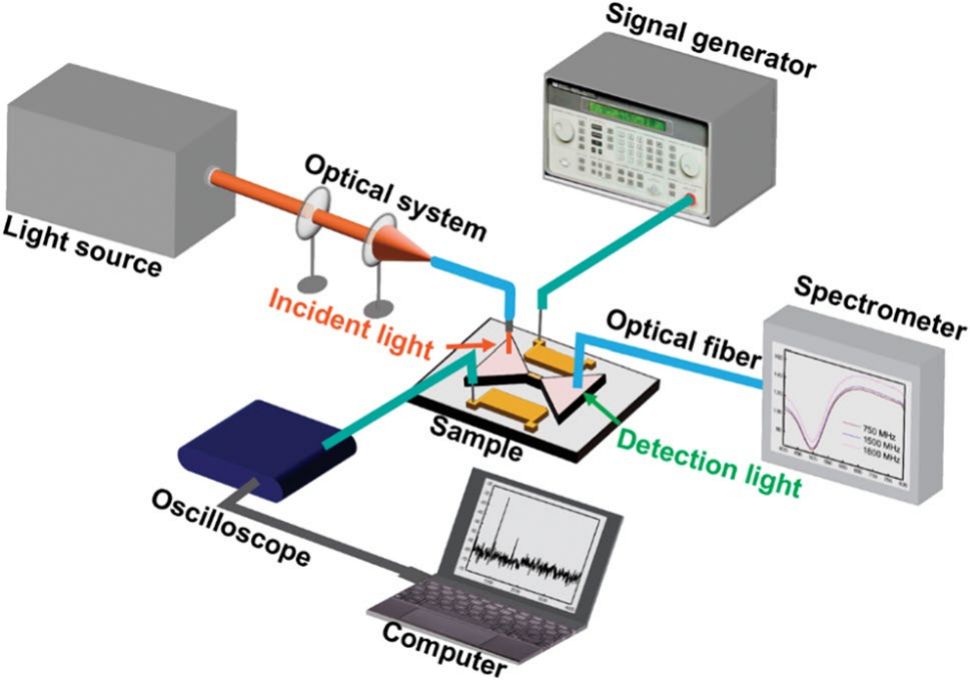
Schematic of the experimental setup for SAW-excited plasmonic waveguide. The optical setup for generating the SPP in the plasmonic waveguide consists of a light source, collimated lenses, optical fibers, and a spectrometer. As for the IDT, an electrical signal generator was employed to generate SAWs. The other IDT was used to detect and convert SAWs to electric signals so that an oscilloscope could analyze the acoustic properties

The measurement setup, shown in Fig. 2, consists of two parts, excitation and detection of surface plasmon, and the generation of the acoustic waves. To excite the surface plasmons, white light generated from a Xenon lamp (ASB-Xe-175) was collimated to an optical fiber and then illuminated on the input port of the waveguide. The incident angle was adjusted for maximum input coupling. The excited surface plasmons propagated along the waveguide and were collected by a spectrometer (Ocean Optics, HR 4000). An AC electric signal was applied to the IDT on the LN substrate to excite the SAWs. The AC swing peak-to-peak voltage and frequency of the AC signal were tuned in the experiment. On the other side of the waveguide, a second IDT was used to detect and convert SAWs (generated from the first IDT) back to electrical signals to monitor SAW properties by an oscilloscope.

## Theoretical conceptualization of SPP and SAW interaction

### Theoretical analysis

**Fig. 3:**
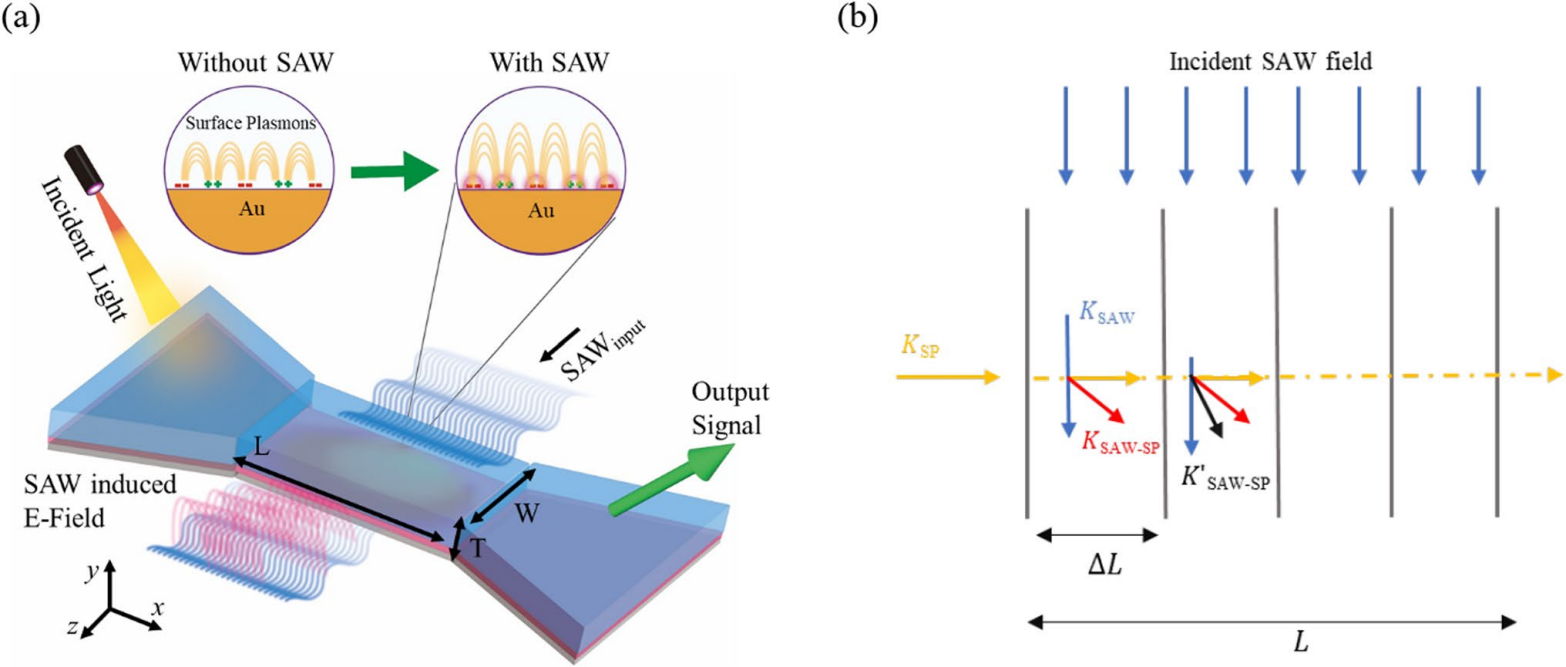
**a** Illustration of the interaction of SAWs with propagating SPP in a plasmonic waveguide. **b** Representation of SAW-SP field interaction, with the surface plasmon wavevector (K_SP_ in yellow line), SAW wavevector (K_SAW_ in blue line), and the diffracted wavevector after interaction (K_SAW-SP_ in red line (in the first $\Delta {\text{L}}$) and $K^{\prime}$_SAW-SP_ black line (in the second $\Delta {\text{L}}$))

The interaction between the SAW and the propagating plasmons in the waveguide is conceptualized and represented in Fig. 3a. The plasmonic wave is generated at the metal–dielectric interface on the xy-plane and propagates along the x-direction. On the other hand, the IDT-generated acoustic wave propagates perpendicular to the plasmonic field, specifically along the z-direction. The interaction between the plasmonic wave and the acoustic wave occurs in a confined region defined by the cross-sectional area of W × L.

When light is incident on the waveguide at the input port, it travels through the Au-SiO_2_ interface and excites SPs that propagate along the channel. Simultaneously, the IDT generates SAW propagating through the material [35]. The SAW leads to a periodic refractive index variation along the z-direction. This phenomenon can be understood by considering the modulation of the refractive index caused by the SAW-induced elastic deformation. The elastic deformation alters the local electron density and distribution of electrons, affecting the effective refractive index of the medium. When the SP propagates along the waveguide, the periodic refractive index variation introduces a cumulative modulation of the plasmonic field.

As shown in Fig. 3b, the plasmonic field, converted from the light field when it travels $\Delta {\text{L}}$ through the Au-SiO_2_ interface, carries a wavevector ${K}_{SP}$. During the propagation along the waveguide, it diffracted by interacting with the SAW field, with the wavevector ${K}_{SAW}$, as expressed by the following equation [36, 37],

1 $${K}_{SAW-SP}={K}_{SP}+{K}_{SAW}$$

where ${K}_{SAW-SP}$ is the wavevector of the diffracted plasmons. Equation 1 can be considered an energy transfer process between the phonons and plasmons. Continuing the process of diffraction in the first segment of $\Delta {\text{L}}$, the interaction of SAW-SP in the second $\Delta {\text{L}}$ is composed of a wavevector of newly generated ${K^{\prime}}_{SP}$, diffracted *K*_*SAW-SP*_ in the first segment, and ${K}_{SAW}$. The newly diffracted plasmon wavevector, ${K^{\prime}}_{SAW-SP}$, illustrated in black line in Fig. 3b is expressed as

2 $${K^{\prime}}_{SAW-SP}={K^{\prime}}_{SP}+{{K}_{SAW}+K}_{SAW-SP}$$

${K^{\prime}}_{SAW-SP}$ indicates a cumulated result of energy transfer from light field and SAW, which suggests a more rapid increase of plasmonic intensity along the waveguide than in the case without SAW.

### Simulation

**Fig. 4:**
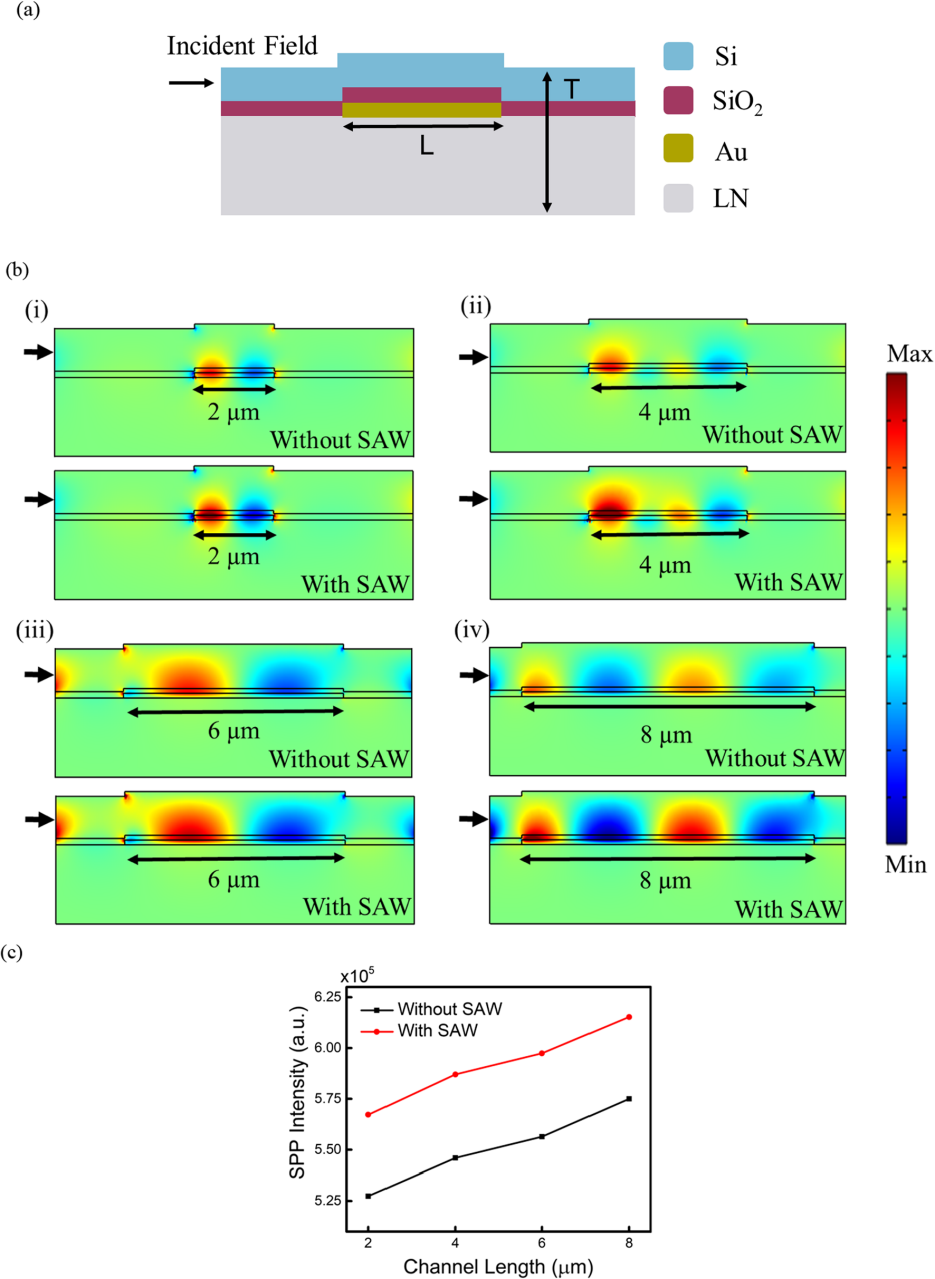
**a** Side-view of the device employed for simulation. **b** Comparisons of simulated SPP intensity distribution with and without the application of SAW excitation. The channel length is (i) 2 µm, (ii) 4 µm, (iii) 6 µm, and (iv) 8 µm. **c** Simulated SPP intensity at various channel lengths with and without SAWs

To visualize the influence of SAWs on plasmonic propagation, we simulated the SPP intensity distribution along the waveguide with various interaction cross-sectional areas. Figure 4a shows the structure for simulation, in which light is incident from the left on the Si layer and L indicates the channel length of propagation. The simulation study was conducted using the radio frequency module of COMSOL Multiphysics 5.6 software. Specifically, the simulation incorporated scattering boundary conditions and user-defined ports to apply the electric field. To facilitate the generation of surface plasmon propagation, we employed Lorentz-Drude approximation through the surface conductivity model and transitional boundary conditions. Furthermore, an additional port was defined to simulate the electric field induced by the acoustic wave. The orientation of this port is set perpendicular to the orientation of the first port. This approach considers that the SAW induces a secondary electric field along its propagation path. The simulated SPP intensity (${\left|E\right|}^{2}, E: mode profile)$ distribution along the waveguide was analyzed by varying interaction cross-sectional areas to gain insights into the influence of SAWs on plasmonic propagation.

The simulated SPP intensity distribution along the waveguide of channel length from 2 to 8 µm is shown in Fig. 4b (i)–(iv). The SPP intensity enhancement across the cross-section was assessed and compared with and without the excitation of SAWs. Figure 4c plots the SPP intensities (${\left|E\right|}^{2}, E: mode profile)$ at the output port. Without SAW excitation, the SPP intensity increases linearly with the guided channel length, with the highest SPP intensity at 8 µm. With SAW excitation, amplification of the SPP intensity is observed for all the channel lengths.

## Experimental results and discussion

### Characterizations of SAWs under various AC electrical signals

**Fig. 5:**
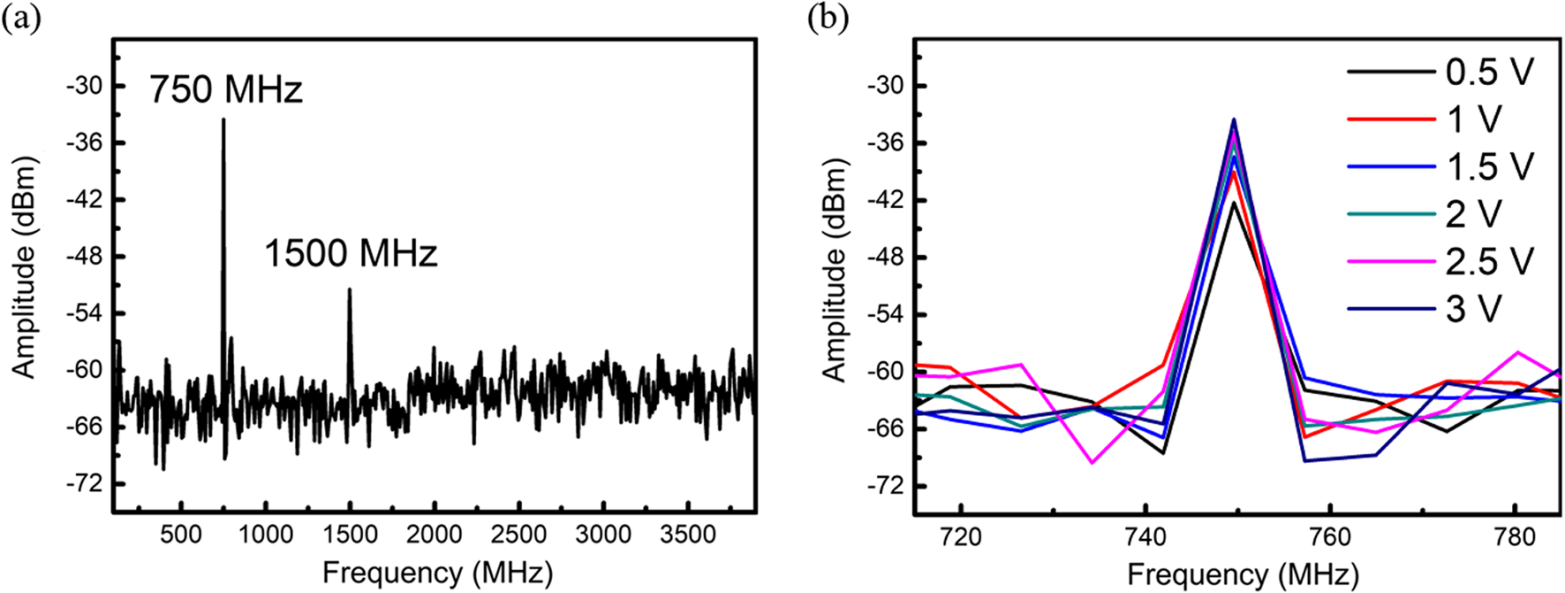
**a** SAW spectral response over frequency variation when 3 V AC electrical signal is applied to the IDT, **b** SAW spectra near 750 MHz under various IDT potentials

We first characterize the acoustic properties of an IDT under AC electrical signals. The structural parameters of the IDT determine the resonant frequencies, such as finger width, finger-to-finger distance, and piezoelectric properties of the substrate [38, 39]. The amplitude of the generated SAW can be varied by tuning the input electrical potential to the IDT. Following the designated IDT geometry described above, we applied a 750 MHz AC electrical signal to the top IDT as in Fig. 2. The corresponding SAW spectral response were detected from the bottom IDT by converting acoustic signals back to electrical. The results are shown in Fig. 5a. The maximum amplitude of the SAW is observed at a fundamental mode frequency of 750 MHz. A second harmonic response at 1500 MHz is also shown. Figure 5b demonstrates SAW amplitudes near the resonant frequency of 750 MHz when the applied peak-to-peak potential is in the range of 0.5 and 3 V. When voltage is applied to a piezoelectric material through IDT, it undergoes deformation due to the inherent piezoelectric effect, transforming electrical energy into mechanical strain. This strain generates shear stress, which translates into an amplified mechanical wave propagating across the material's surface in the context of SAW. Therefore, as the applied voltage increases, the piezoelectric conversion intensifies, leading to a larger SAW response characterized by enhanced wave strength and propagation [34, 39].

### Plasmon propagation along the waveguide (without SAWs)

**Fig. 6:**
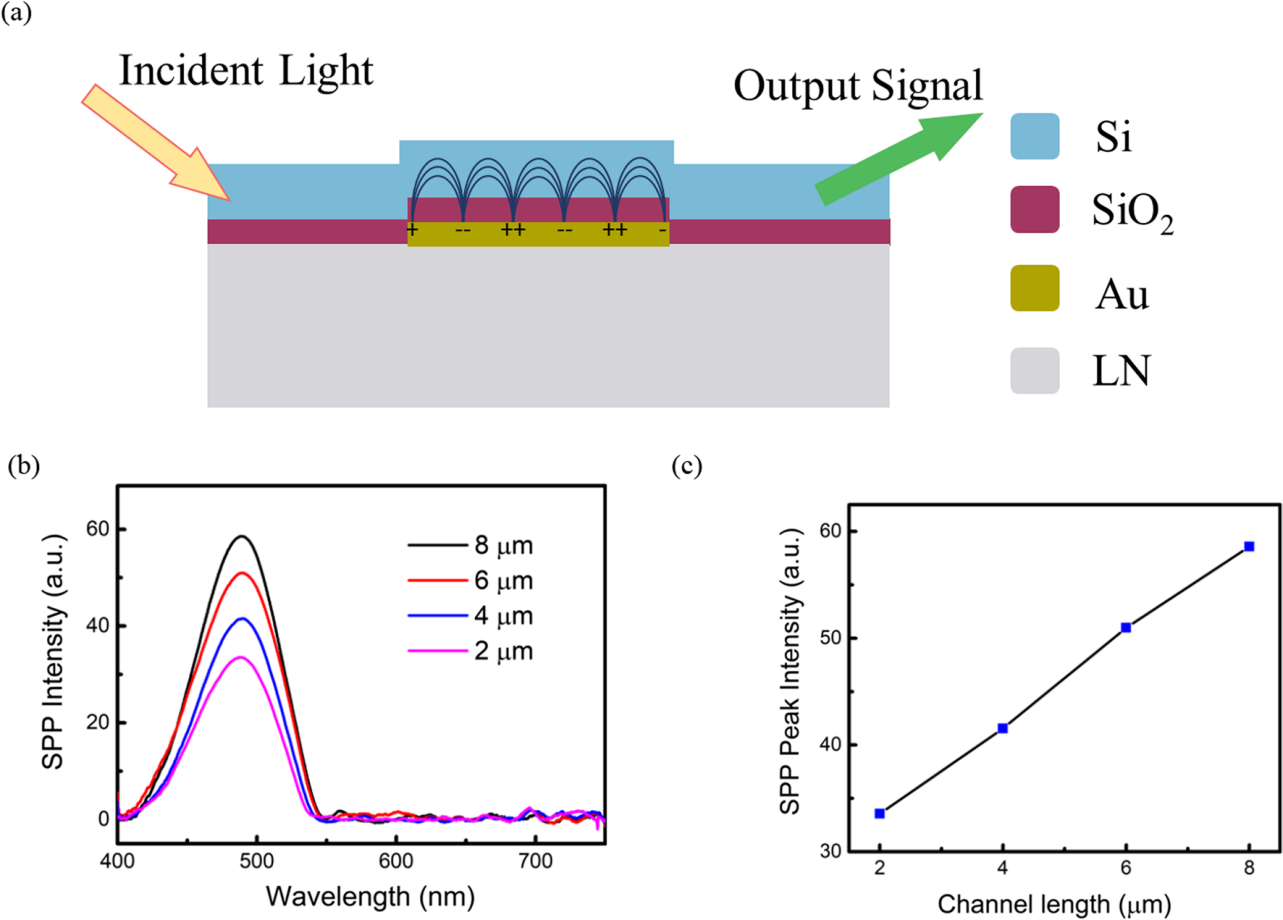
**a** Illustration of the generation and propagation of SPP along the waveguide with the light incident. **b** Experimental observation of the spectral plasmonic responses with a channel length of 2, 4, 6, and 8 µm. The SPP intensity was extracted without applying SAWs. **c** The plasmonic intensity was measured at different channel lengths

Plasmonic wave propagation is studied by shining white light on the input port. As shown in Fig. 6a, when the incident light propagates through the waveguide slab, several optical modes penetrate the SiO_2_ layer and generate plasmons that travel in the Au layer. The resonant frequency of the waveguide's plasmonic response and its plasmonic strength can be determined from the absorption of the output optical spectrum. The plasmonic responses at the various channel lengths are plotted in Fig. 6b. The peak absorption at the wavelength of 495 nm is associated with the collective oscillation of plasmonic electrons in the Au layer.

The propagation length along the waveguide determines the probability of photon absorption and plasmon generation. The incident light propagated along the waveguide experiences material-absorption loss, scattering loss, and the conversion of optical intensity to plasmons. Within a certain waveguide length, SPP strength increases with the propagation length [40]. It can be understood by considering the electron population in the plasmonic state in the surface area of the Au layer. As light is propagated along the channel, the number of excited plasmons that transfer to SPP mode increases, resulting in the net amplification of SPP. Hence, in Fig. 6c, the output plasmonic response increases with the channel length, suggesting that the conversion of photons to plasmons is strong enough to compensate for the propagation loss. However, further waveguide length increases inevitably leads to decreased plasmonic strength [40].

### Plasmonic wave interaction with SAWs in the waveguide

**Fig. 7:**
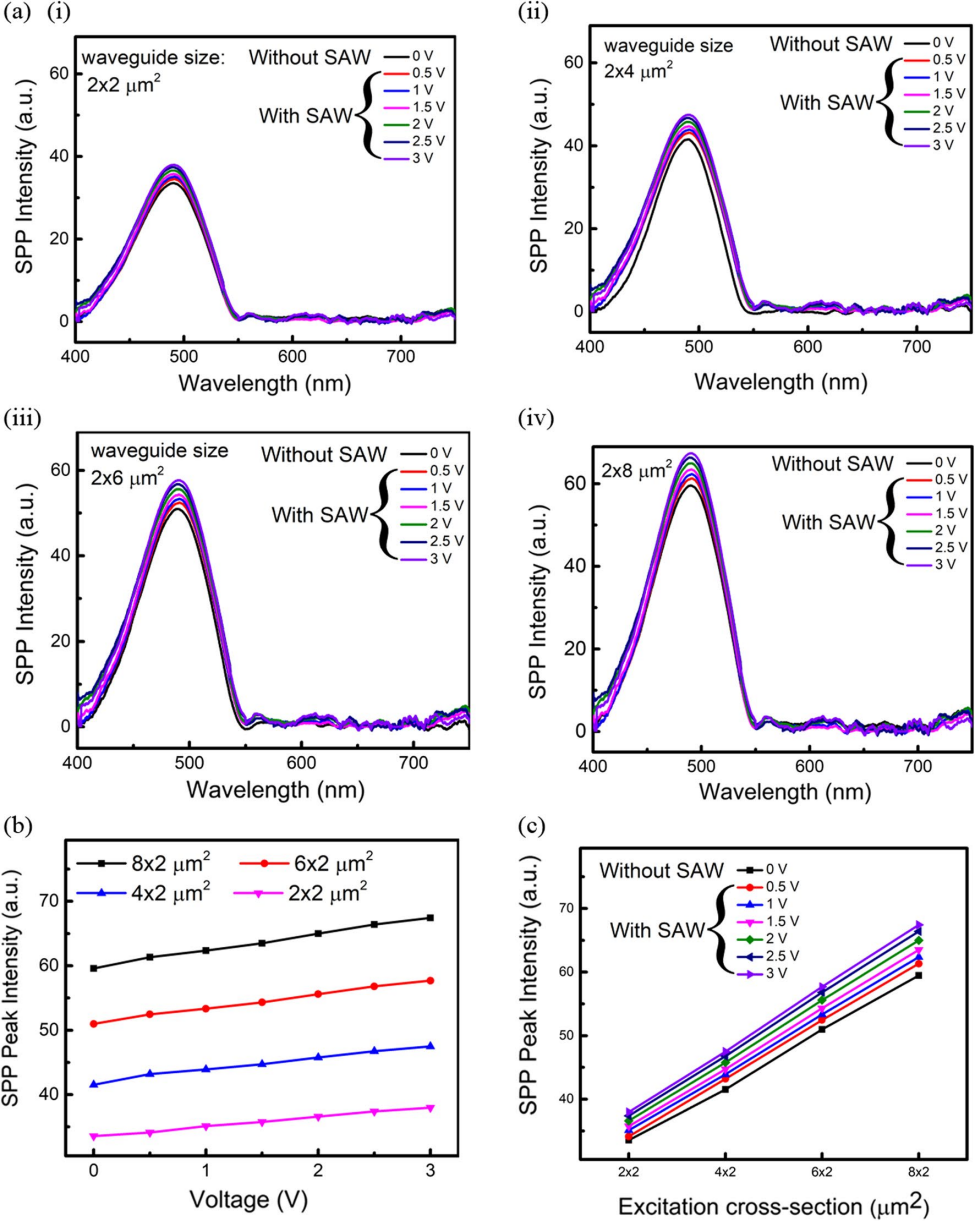
**a** Comparisons of spectral plasmonic responses with (0.5–3 V) and without (0 V) the application of SAW excitation. The excitation cross-section is (i) 2 × 2 µm^2^, (ii) 2 × 4 µm^2^, (iii) 2 × 6 µm^2^, and (iv) 2 × 8 µm^2^. **b** Peak intensity variations of SPP under different bias voltages on the IDT. 0 V indicates the measurements without SAW. **c** Peak intensity of SPP spectra for different excitation cross-sections with (0.5 ~ 3 V) and without (0 V) the presence of SAW

The effect of SAW on propagating SPP is experimentally studied by exciting SAWs perpendicular to the direction of SPP propagation in the waveguide, as represented in Fig. 3. We extracted the SPP intensity under the influence of various SAW magnitudes and frequencies. Figure 7a (i)–(iv) show the SPP spectra for various excitation cross sections and applied potentials. Figure 7b, c summarize the correlation between SPP peak intensity, peak-to-peak swing voltage, and excitation cross section. The applied voltage of 0 V in Fig. 7 represents the measurements without SAW. The SPP intensity at the waveguide output increases with the applied potential on the IDT (and thus the increase of SAW magnitude) and excitation cross section. It suggests that the plasmonic waveguide behaves as an external gain medium under SAW excitation. The results are attributed to creating a stronger phononic oscillation at a higher IDT potential in the surrounding media of the waveguide [41, 42]. The mechanical disturbance excites more surrounding phonons to a high-energy state. When encountering plasmons, the excess of phonons alters or slows down the energy relaxation of the plasmon–phonon coupling. Thus, the corresponding electron population density of the plasmonic state increases, leading to higher plasmonic peak intensity. The coupling can be further understood from Eq. (1) and (2), which describe the momentum (or wavevector) transfer between SPP and SAW.

**Fig. 8:**
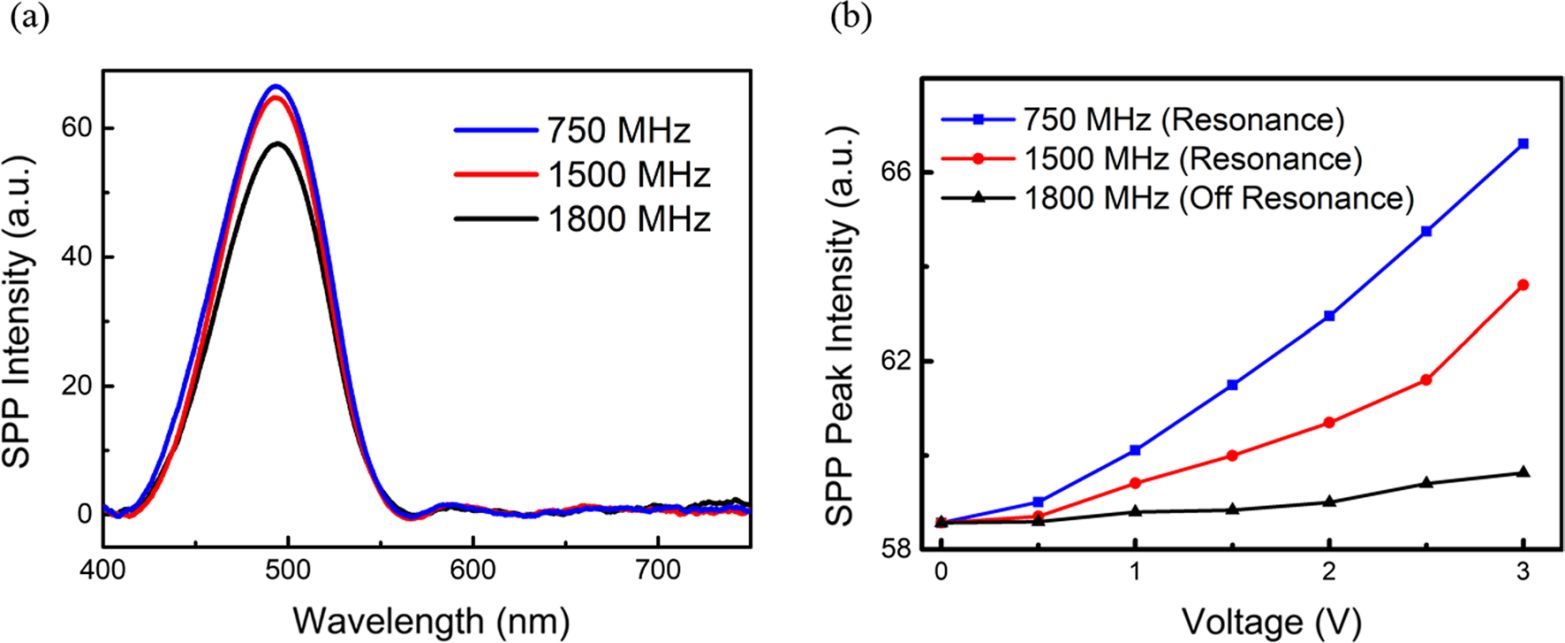
**a** SPP spectra at various AC swing frequencies. The peak-to-peak voltage exerted on the IDT is 3 V, **b** Peak plasmonic intensity v.s. applied swing voltage at SAW resonant and off-resonant frequencies. The excitation cross-section of the waveguide was kept at WxL (2 × 8) µm^2^ for all the results above

The plasmonic spectral response at various SAW frequencies is shown in Fig. 8a. The peak-to-peak AC swing potential on the IDT is 3 V. Since the SAW strength is maximum at its fundamental resonance frequency of 750 MHz, momentum transfer is maximum at the resonance frequency. As for the case of the second-order resonant mode, 1500 MHz, the plasmonic intensity is less than that at the fundamental dominant mode. On the other hand, when the IDT is off the resonance, e.g., at 1800 MHz, the SPP intensity is lower than those at resonant modes. The potential-dependent SPP peak intensities at different SAW frequencies are summarized in Fig. 8b. At 1800 MHz, the variation of SPP intensity with the IDT potential is less noticeable.

### Variation of plasmonic response on SAW excitation cross-section

**Fig. 9:**
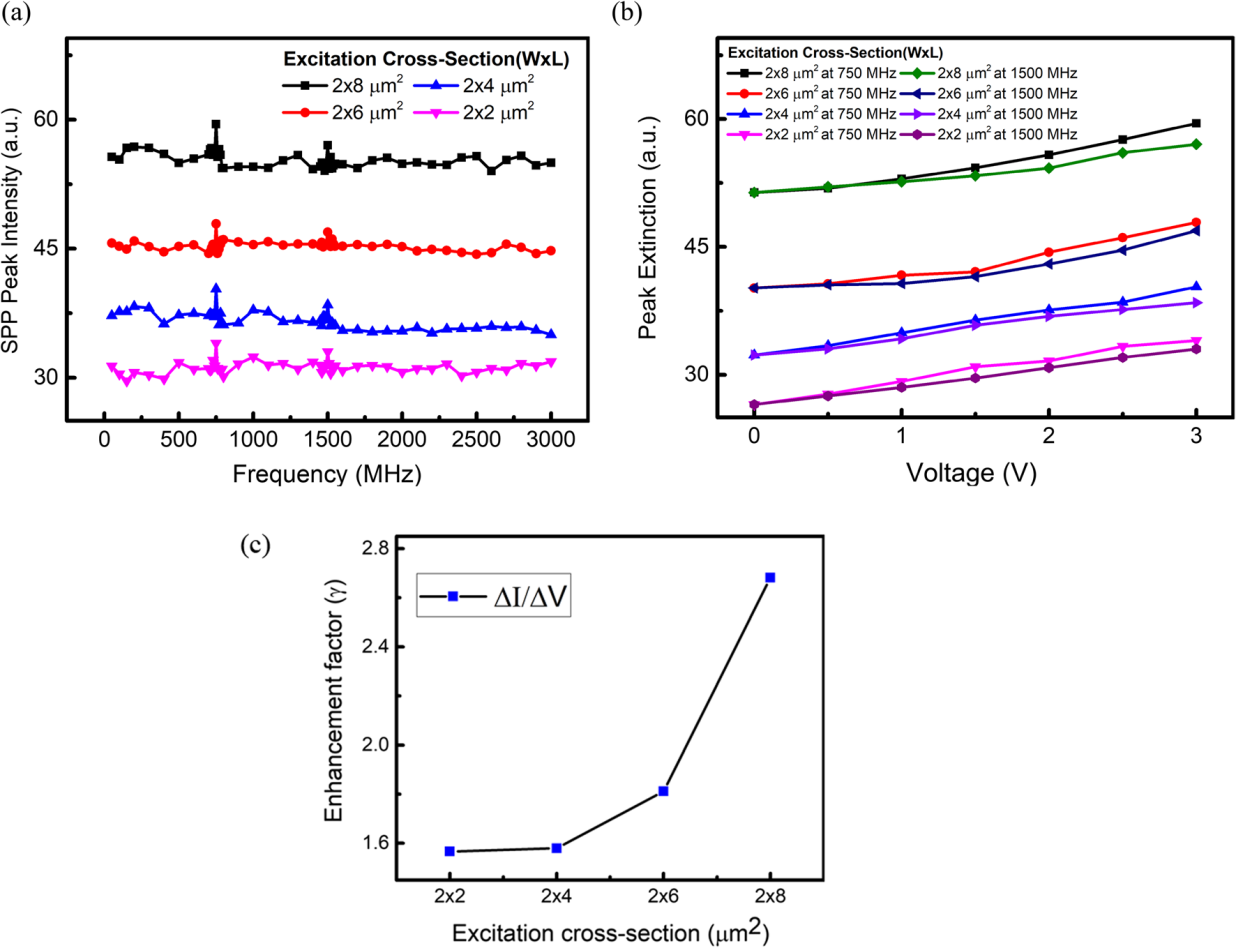
**a** Plasmonic intensity at various SAW frequencies and excitation cross sections. The applied potential to the IDT is 3 V. **b** Plasmonic peak intensity at various IDT potentials. Cross sections ranging from 2 × 2, 2 × 4, 2 × 6, and 2 × 8 µm^2^ are compared. **c** Relation between excitation cross-section area to SAW-induced plasmonic enhancement factor, $\gamma$

The plasmonic response of SAW excitation on different waveguide cross-sections is shown in Fig. 9a. The applied IDT potential is 3 V, and the swing frequency is 750 MHz. For a fixed excitation cross-section, the plasmonic peak intensity at the fundamental SAW resonant frequency (750 MHz) is greater than that at the second order (1500 MHz), mainly due to a large SAW amplitude at 750 MHz. The variation of applied IDT potential to plasmonic intensity at 750 and 1500 MHz is plotted in Fig. 9b at various excitation cross-sections. The difference of SPP peak intensity between fundamental and second order resonant frequencies is more pronounced at a larger voltage swing.

We define an enhancement factor, $\gamma$, as the ratio of the change in the plasmonic intensity (${\Delta I=I}_{SAW-SP}-{I}_{SP}$) (before and after the application of SAW) to the swing voltage, $\Delta V$. That is, $\gamma$ represents the SPP intensity gain per unit voltage.

3 $$\gamma =\frac{\Delta I}{\Delta V}$$

$\gamma$ can be used to understand the propagation characteristics of the waveguide under external SAW excitation. The plasmonic signal intensity at the output of the waveguide mainly depends on ${I}_{SP}$ and $\gamma$. Figure 9c represents the relationship between the $\gamma$ and excitation cross-section at the applied SAW potential of 3 V at 750 MHz. The gain increases more pronouncedly with a larger excitation cross-section for the device architecture. When the excitation cross section is large, the incident light can efficiently excite SPPs, and their damping due to the scattering and absorption processes becomes significant. The modulation of the plasmonic field coupled with SAWs at a large excitation cross-sectional area can help overcome the damping of the SPPs and enhance the overall plasmonic gain.

Moreover, we define the SPP intensity gain factor, $\alpha$, as the SAW-coupled plasmonic output intensity (${I}_{SAW-SP}$) ratio to the plasmon signal (${I}_{SP}$) at the output without SAW.

4 $$\alpha =\frac{{I}_{SAW-SP}}{{I}_{SP}}$$

The gain factor is alternatively expressed in logarithmic decibel as,

5 $$\alpha \left(dB\right)=20 log\left(\frac{{I}_{SP-SAW}}{{I}_{SP}}\right)$$

Table 1 shows the SPP intensity gain for various waveguide cross sections. Though the SPP peak intensity increases with the waveguide length for the cases with and without applying SAW excitation, the gain factor is less dependent on the waveguide propagation length. It implies that the perpendicular acoustic interaction with the plasmonic wave is uniformly distributed. It also confirms that the model describing the interactions of both waves in Fig. 3b is independent of the cross-sectional areas.

**Table 1:** Gain factor, $\alpha$, of the plasmonic waveguides with different SAW excitation cross sections

SAW excitation waveguide cross sections (${\mu m}^{2}$)	$\alpha =\frac{{I}_{SAW-SP}}{{I}_{SP}}$	$\alpha \left(dB\right)=20 log\left(\frac{{I}_{SP-SAW}}{{I}_{SP}}\right).$
2 × 2	1.13	1.07
2 × 4	1.12	0.98
2 × 6	1.13	1.08
2 × 8	1.13	1.08

Overall, the above observations confirm the feasibility of fabricating a novel SAW-based on-chip amplifier for hybrid plasmonic waveguides, which can effectively manipulate the plasmonic output signal by tuning the SAW waveforms. A significant hurdle in current plasmonic waveguide technology is improving the plasmonic propagation with decent confinement. Integrating plasmonic waveguides with IDTs offers the potential for post-fabrication enhancement and modulation of plasmonic response, providing a distinct advantage for nanoscale guiding technology. In Table 2, we compare various plasmonic waveguides and parameters of concern. The potential applications are also listed. For our devices, the enhanced intensity achievable through SAW-actuated plasmonic waveguides confers a series of advantages, rendering them particularly suitable for the development of more efficient signal amplification or repeating devices. Examples of such device applications include plasmonic amplitude and phase modulators and plasmonic repeaters for on-chip plasmonic waveguide communication.

**Table 2:** Comparison of various plasmonic waveguide structures and the corresponding applications

Waveguide structure	Parameters of concern	Potential applications	Reference
Plasmonic slot waveguide	Intensity contrast ratio: 24 dB, (For Logic gates)	Logic gates	[43]
Hybrid integrated plasmonic photonic waveguides (Gold nanorod)	Light coupling: 9.7%, Signal to Noise: > 20 dB, Sensitivity: ∼ 250 nm/RIU	Sensing and spectroscopy	[44]
Monolithic plasmonic waveguide	Amplitude modulation: 10 dB, Insertion loss: < 1 dB	Plasmonic circuits	[45]
Hybrid photonic-plasmonic waveguides	Propagation length: 7.2 mm, Propagation loss: 0.6 dB/mm, Mode size: 7.7 μm	CMOS-compatible materials, and photonic circuits	[46]
CMOS copper plasmonic waveguides	Propagation length: > 40 $\mu m$, Mode size: < ${\lambda }^{2}/50$	Optical interconnects	[47]
SAW actuated Plasmonic waveguide	Amplitude enhancement: 13%, Signal gain: 1.08 dB, (Waveguide size: 2 × 8 $\mu {m}^{2}$ at 3 V)	Plasmonic amplifiers, plasmonic signal repeaters, amplitude modulators	This Work

## Conclusion

A SMIS (semiconductor–metal-insulator-semiconductor) based hybrid plasmonic waveguide is fabricated with a unique SAW integrated structure. The IDT-integrated plasmonic waveguide provides an on-chip gain media for propagating SPP with a gain factor of around 1.08 dB at 3 V. The amplitude and resonant frequency of SAWs play a vital role in deciding the plasmonic amplification in the waveguide. At the primary resonant frequency of IDT, the gain in plasmonic response is maximum. Increasing the input voltage of IDT amplifies the plasmonic signal. Therefore, the voltage tuning of plasmonic propagation is possible with desired signal level. The increasing channel dimensions result in better SPP generation (for microscale light injection) but increase the decay constant (nanoscale light injection). Therefore, using SAW integration improves the decay constant for more extended propagation. The on-chip plasmonic enhancement can advance a wide range of existing and future plasmonic devices, such as biosensors with high sensitivity and plasmonic waveguides with long-distance propagation.

## Data Availability

Data underlying the results presented in this paper are not publicly available at this time but may be obtained from the authors upon reasonable request.
